# Trends of the mental health of the Hungarian adult population between 2010-2023

**DOI:** 10.1192/j.eurpsy.2024.266

**Published:** 2024-08-27

**Authors:** K. Kósa, É. Bíró

**Affiliations:** ^1^Department of Behavioural Sciences; ^2^Department of Public Health and Epidemiology, University of Debrecen, Debrecen, Hungary

## Abstract

**Introduction:**

Several data have been published in the past decade on the mental health of the Hungarian population by different research teams but less information is available about the trends of mental status of the population based on comparable research methods.

**Objectives:**

Our aim is to provide data on the time trends of mental status in Hungary using comparable methodology.

**Methods:**

Four cross-sectional mental health surveys of the adult Hungarian population were designed by the authors; data collection was carried out by an opinion polling company between 2010 and 2023. Representative samples were selected by multistage stratified cluster sampling and weighted for analysis. Self-filling questionnaires were used to collect information on demographic data and socioeconomic status; validated scales were used to assess pathological distress (GHQ) and sense of coherence (SOC).

**Results:**

Distribution of the respondents by permanent residence, age and sex in all surveys reflected that of the adult population of the country. The proportion of adults struggling with severe psychological distress approximately halved from 14.53% in 2010 to 6.78% in 2019 showing a significantly improving trend (<0.001) with higher proportions of women being severely stressed compared to men. Sense of coherence, a measure of psychological resilience increased by 3.43 points from 2010 to 2019 (p<0.001), also reflecting a significantly improving trend without gender difference. However, the last survey of 2023 showed significant worsening with 18.85% of adults struggling with pathological distress, and a dramatic decrease in sense of coherence (-13.64 points).

Educational level and social support were found to be consistent and significant determinants of mental health.

**Conclusions:**

Mental health status improved among Hungarian adults in the past decade up until 2019 but the trend turned into the opposite by 2023. Further research is warranted to uncover the underlying causes of the latest changes.

**Disclosure of Interest:**

None Declared

